# The Effect of Chemical Environment and Temperature on the Domain Structure of Free‐Standing BaTiO_3_ via In Situ STEM

**DOI:** 10.1002/advs.202303028

**Published:** 2023-08-21

**Authors:** Tamsin O'Reilly, Kristina M. Holsgrove, Xinqiao Zhang, John J. R. Scott, Iaro Gaponenko, Praveen Kumar, Joshua Agar, Patrycja Paruch, Miryam Arredondo

**Affiliations:** ^1^ School of Mathematics and Physics Queen's University Belfast Belfast BT7 1NN UK; ^2^ University of Glasgow Glasgow G12 8QQ UK; ^3^ Department of Mechanical Engineering and Mechanics Drexel University Philadelphia PA 19104 USA; ^4^ DQMP University of Geneva Geneva 1211 Switzerland; ^5^ Shared Instrumentation Facility Colorado School of Mines Golden CO 80401 USA

**Keywords:** BaTiO_3_ free‐standing film, chemical environment, in situ heating STEM, domains

## Abstract

Ferroelectrics, due to their polar nature and reversible switching, can be used to dynamically control surface chemistry for catalysis, chemical switching, and other applications such as water splitting. However, this is a complex phenomenon where ferroelectric domain orientation and switching are intimately linked to surface charges. In this work, the temperature‐induced domain behavior of ferroelectric‐ferroelastic domains in free‐standing BaTiO_3_ films under different gas environments, including vacuum and oxygen‐rich, is studied by in situ scanning transmission electron microscopy (STEM). An automated pathway to statistically disentangle and detect domain structure transformations using deep autoencoders, providing a pathway towards real‐time analysis is also established. These results show a clear difference in the temperature at which phase transition occurs and the domain behavior between various environments, with a peculiar domain reconfiguration at low temperatures, from a‐c to a‐a at ≈60 °C. The vacuum environment exhibits a rich domain structure, while under the oxidizing environment, the domain structure is largely suppressed. The direct visualization provided by in situ gas and heating STEM allows to investigate the influence of external variables such as gas, pressure, and temperature, on oxide surfaces in a dynamic manner, providing invaluable insights into the intricate surface‐screening mechanisms in ferroelectrics.

## Graphical abstract

Ferroelectric surfaces can enable dynamic surface chemistry control due to their polar nature and reversible switching. This study investigates temperature‐induced domain behavior in free‐standing ferroelectric films under various gas environments using in situ scanning transmission electron microscopy and deep autoencoders. Real‐time analysis of domain structure under different environments provides invaluable insights into intricate surface‐screening mechanisms, responsible for surface chemistry control.

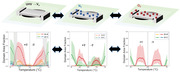

## 1 Introduction

Ferroelectrics are polar materials characterized by their spontaneous polarization that can be reversibly switched by applying an external electric field. The resulting domain structure, and switching dynamics, are determined by electrostatic boundary conditions. In particular, the compensation of polarization‐induced surface charges is a key factor in stabilizing ferroelectricity. In typical ferroelectric devices, charge compensation is achieved through the screening provided by the metallic electrodes, where domains will be formed to minimize the depolarization field energy. However, any deviation from this ideal scenario, including the presence of surface charges such as charged ionic species adsorbed from the atmosphere or vacancies,^[^ ^1^, ^2^ ^]^ will significantly affect the screening dynamics and ferroelectric properties. For decades, the polar surfaces of ferroelectrics have been considered extremely interesting entities, where the possible controlled exchanges between the chemical species in a chemical environment and polarization can be exploited to tailor surface reactivity for electrochemical, catalytical,^[^ ^3^, ^4^, ^5^, ^6^, ^7^, ^8^, ^9^ ^]^ and other energy harvesting applications.^[^ ^4^, ^10^, ^11^, ^12^ ^]^ Importantly, the relationship between the chemical environment (atmospheric adsorbates) and ferroelectric polarization is two sided: i) the (polar) domains can significantly influence the local surface potential and surface free energy, thus, influencing the chemistry at the ferroelectric's surface, and ii) the chemical environment can provide charged species which can be used to control the polarization orientation and domain switching.^[^ ^1^, ^13^, ^14^ ^]^ It is crucial to understand both aspects of this relationship to effectively control the properties and behavior of ferroelectric materials for their development towards novel applications aimed at electrochemistry and controllable surface chemical reactions,^[^ ^15^ ^]^ which have shown promising for applications such as water splitting, and piezo‐catalysis.^[^ ^16^, ^17^, ^18^, ^19^, ^20^ ^]^ This is a complex phenomenon and the current understanding of this remains an open theoretical and experimental challenge. Another important aspect to consider when investigating surface effects on oxides is temperature. Morozovska et al. demonstrated theoretically that temperature plays a non‐trivial effect on the polarization and nature of the surface charge.^[^ ^21^ ^]^ Thus far, experimental efforts attempting to disentangle the role of surface chemistry, polarization direction, and surface structure have been dominated via X‐ray scattering,^[^ ^13^ ^]^ X‐ray photoelectron spectroscopy (XPS),^[^ ^22^, ^23^ ^]^ and low energy electron diffraction (LEED),^[^ ^24^, ^25^ ^]^ most of them on bulk. To date, a limited number of publications have reported on the simultaneous imaging of the domain structure under different chemical environments, such as Kim et al., who reported chemical switching in BiFeO_3_ thin films using piezoresponse force microscopy (PFM).^[^ ^14^ ^]^

Here, we investigate the temperature‐dependent behavior of ferroelectric‐ferroelastic domains in free‐standing BaTiO_3_ (BTO) films under distinct atmospheres via in situ scanning transmission electron microscopy (STEM) techniques. The term 'free‐standing' refers to the fact that, unlike traditional epitaxial heterostructures, the ferroelectric material is not attached to a substrate. By using free‐standing films, we eliminate substrate‐related effects such as rigid clamping, chemical gradients, etc. Additionally, free‐standing ferroelectrics have recently gained great interest as they exhibit interesting effects that differ from the bulk.^[^ ^26^, ^27^, ^28^, ^29^ ^]^

In situ TEM gas cells have been successfully used to investigate catalysis^[^ ^16^, ^17^ ^]^ and other phenomena. However, to our knowledge, this is the first instance of in situ gas STEM being used to study ferroelectric domains and phase transformations under controlled environments. We complemented the STEM observations with in situ heating PFM under ultrahigh vacuum (UHV) conditions. Importantly, we also establish an automated pathway to statistically disentangle and detect changes in the domain structure using deep autoencoders, providing a pathway toward real‐time analysis. In summary, we offer valuable insight via direct domain imaging, into the delicate link between key ferroelectric characteristics and the chemical environment, reporting clear variations on the phase transition temperature (*T*_C_), domain size, and periodicity presented. This direct visualization demonstrates in situ gas STEM as a novel technique to explore the effect of external variables (gas, pressure, and temperature) on oxide surfaces in a dynamic manner, particularly, to investigate surface‐screening mechanisms in ferroelectrics.

## 2 Results and Discussion

To thoroughly discuss the results presented here, it is essential to first introduce i) the sample configuration, along with the in situ TEM system, and ii) the image segmentation method used to calculate the domain area fraction.

### 2.1 In Situ STEM Set Up

**Figure 1:**
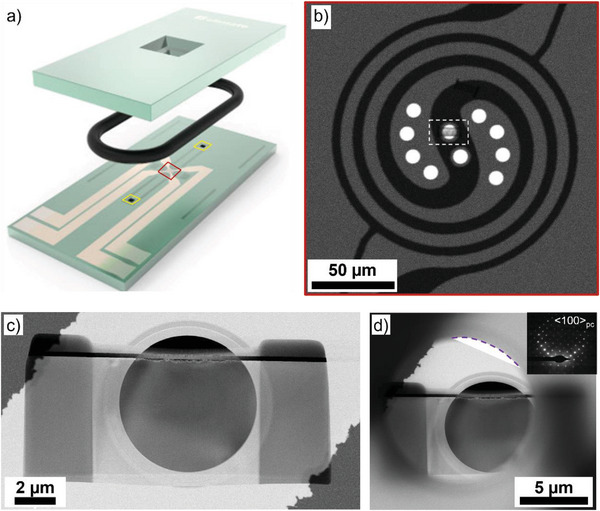
Overview of the in situ holder setup. a) Schematic representation of the DENSsolutions Climate system with the top and bottom MEMs chips forming the nanoreactor (NR), adapted from,^[^ ^30^ ^]^ the central red square indicates the sample area shown in b) and the yellow squares indicate the inlet, and outlet gas ports, b) representative HAADF‐STEM overview of the resistive heating (spiral) with the electron transparent windows (bright circles) displaying the position of a lamella in the central window, marked by the dotted square. HAADF‐STEM overviews of BaTiO_3_ lamellae placed across the electron transparent window at RT in (c) an open cell configuration (without the top chip) and d) positioned within the full assembled NR (top and bottom chips). The purple curved dotted line indicates the position of the top UHR chip. The electron diffraction pattern of the lamella is shown as an inset, indicating its proximity to the <100>_pc_ zone axis.

In situ heating and controlled gas flow were performed on a DENSsolutions Climate system, as shown schematically in **Figure** **1**. Lamellae of ≈150 nm thickness were prepared from BaTiO_3_ (BTO) and LaAlO_3_ (LAO) single crystals by focused ion beam (FIB) and transferred to a DENSsolutions Climate MEMs bottom chip with the lamellae positioned as shown in Figure 1 and Figure S1 (Supporting Information) and detailed in the Experimental Section. Heat cycles were performed from room temperature (RT) to 250 °C, and back down to RT, at 1 °C s^−1^. High‐angle annular dark‐field (HAADF) and bright‐field STEM (BF‐STEM) images were acquired every 5 °C. First, an open cell configuration (without the top MEMS chip, Figure 1c) was used to heat the sample under the TEM's UHV and afterward, the full nanoreactor (NR) was assembled using an ultra‐high resolution (UHR) MEMs top chip for the introduction of N_2_, followed by 20% O_2_/Ar (of 99.999% purity), Figure 1d. UHV conditions were restored by evacuating the NR. For each gas, the system was flushed for 5 min before the pressure and flow were set to 900 mbar and 0.3 mL min^−1^, respectively, and, both parameters were stabilized prior to in situ heating.

### 2.2 Image Segmentation

**Figure 2:**
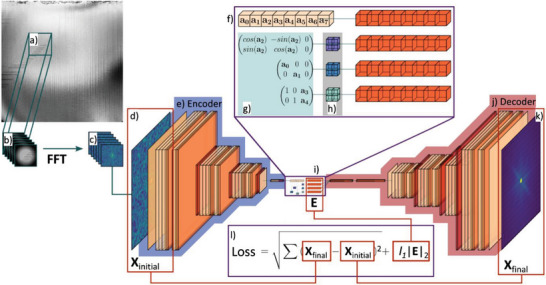
Image segmentation sequence. a) Sample sliding windows, b) apply Hann filter to windows, c) apply FFT to windows, d) initial input stack (1 × 128 × 128 images), e) the Encoder expands images into 128 × 128 × 128 convolutional channels and performs normalization, ReLu, and pooling. f) A 8 pixel feature vector is produced by Encoder. g) Rotational, Scaling, and Translational affine matrices created from the first 5 pixels of the feature vector. h) Rotational, Scaling, and Translational affine grids sampled from each affine matrix. i) Stacked final embedding from original feature vector and flattened affine grids from step h. j) The Decoder mirrors the Encoder and attempts to regenerate input. k) Generated stack of 1 × 128 × 128 images. l) Loss function of the mean squared error between the input and generated image, and embedding intensity multiplied by scaling coefficient *l1* is used for backpropagation and turning model weights.

**Figure 3:**
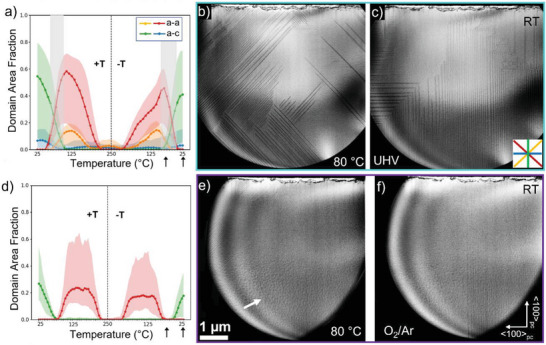
Domain microstructure during heat cycling, at 1°C s^−1^, for UHV and 20% O_2_/Ar (900 mbar, 0.3 mL min^−1^). (a and d) are the relative domain area fraction observed for the full heating cycle, RT –250 °C – RT, for UHV and under the 20% O_2_/Ar, +T and −T indicate heating and cooling, respectively. The gray‐shaded areas in a) highlight the temperatures at which a‐a and a‐c domains coexist and the color‐shaded areas indicate the uncertainty in the domain area fraction. BF STEM images displaying the domain structure in the cool‐down stage, at 80 °C (b and e) and RT (c and f), for UHV and 20% O_2_/Ar, respectively. These reference temperatures are marked by black arrows in (a and d). The inset in (c) indicates the orientation of the domain walls color coded in (a and d). The black circular contour around the sample in the BF STEM images indicates the bottom and top chips from the NR. The white arrow in (e) indicates the mottled pattern developed when the sample is exposed to 20% O_2_/Ar.

Bright‐field STEM images were segmented and analyzed using a three‐part pipeline, where deep learning disentangles the bias caused by sample warping. During preprocessing, the images were cropped, and Gaussian background subtraction was applied. Then, the Pycroscopy package was used to sample (128,128) sized sliding windows and apply a Hanning window and Fast Fourier Transform (FFT), shown in **Figure** **2**a.^[^ ^31^ ^]^ Finally, a logarithm is applied, and the large values are reduced. The preprocessed data is a stack of (128,128) windows of length *T•n•n*, where *T* is the number of temperatures sampled and *n* is the number of windows sampled along each side of the image. Applying FFT to windowed images provides localized information about periodicity and reduces bias caused by sample warping. Next, the FFT dataset runs through a model inspired by the joint rotationally‐invariant variational autoencoder, where input is compressed into a latent space with an additional discrete rotation representation, then reconstructed to compare to the original image.^[^ ^32^, ^33^, ^34^ ^]^ The model used in this article disentangles the input datasets scaling, rotational, and translational elements and appends them to the latent representation. The encoder receives a batch of (128,128) images, which are down sampled and flattened to a feature vector with 8 points, as shown in Figure 2b. The feature vector is then used to construct three affine matrices, with the first two points corresponding to the x and y scaling, the third point to the rotation angle, and the fourth and fifth points to the x and y translation. The eight features and selected affine transformations are shown in Video S1 (Supporting Information). The scaling, rotation, and translation matrices were then used to generate three respective affine grids with shape (2,2,2), and the final latent representation concatenates the feature vector and affine grids. Finally, the feature vector and spatial transformer grids are flattened and used to reconstruct the original input. The model parameters are tuned during training using the loss function shown in Figure 2e. The Mean‐Squared‐Error (MSE) between the input and reconstructed images tests the latent representation for completeness, while L1 Regularization encourages sparsity and prevents overfitting. After training with data from one environment, the model can be tuned via transfer learning to fit data from other environments. In the final step, the feature vectors and affine matrices from all samples are reshaped to eight (*n*,*n*) images (see Supporting Information). These identify domains, sample warping, and contamination, embedding channels [0,4,6,7] in vacuum, Channel 3 in all environments, and Channel 4 in oxygen, respectively. Then the relative areas shown in **Figure** **3** are calculated by averaging the binary mask generated using the Otsu threshold. The error bars are similarly generated by averaging the binary mask generated at one standard deviation above and below the Otsu threshold. For more details, see Figures S2 and S3 (Supporting Information).

### 2.3 Ultra‐High Vacuum (UVH) Environment

Heating under UHV is the most common condition for in situ heating TEM experiments, under which oxygen vacancies are expected to form given that for oxides in general, the vacuum presents a reducing environment during annealing. Thus, as a first instance, UHV provides a reference environment and point of comparison of the domain behavior with other environments here investigated.

The complete domain evolution as a function of temperature is shown in Figure 3a, displaying the relative area fraction of the domains present during the full annealing cycle (from RT to 250 °C and back to RT, at 1°C s^−1^). Four domain wall orientations or variants are identified, referred to as pairs of a‐a and a‐c domains,^[^ ^35^ ^]^ marked by the color scheme shown in the inset of Figure 3a,c. At RT, the sample exhibits a dense domain structure of predominantly fine needle domain walls projected along <100>_pc_, a‐c domains, spanning across the whole lamellae. As the sample is heated, the domain wall mobility increases rapidly, with domains moving (retracting) at temperatures as low as 30–40 °C. Notably, upon heating, the a‐c domains decrease until they disappear at ≈60 °C (blue and green profiles, in Figure 2a). From ≈60 °C, domain wall bundles projected along the <110>_pc_ axis, namely a‐a domains (red and orange profiles in Figure 3a), start to appear. These become the dominant configuration up to ≈190 °C where the contrast decreases, and above this temperature, no domains are observed. Although not as precise as other bulk techniques used to measure T_C_ in bulk, the lack of contrast here observed is an indication of the material undergoing the expected phase transition into the cubic phase, corresponding to the disappearance of the symmetry‐breaking tetragonal distortion, similar to previous reports on lamellae BTO in situ heating.^[^ ^36^, ^37^ ^]^ Upon cooling, the sample displays a hysteretic behavior as a function of temperature, indicative of a first‐order phase transition.^[^ ^38^, ^39^ ^]^ The domain wall variants are present in the same temperature range and with almost identical area fractions as when heated up. The a‐a domains are dominant at high temperatures (Figure 3b) and the a‐c domains are the principal domain wall variant present at low temperatures, with a few “sawtooth” domains found in the top right corner (Figure 3c). Interestingly, there is a temperature range at which a‐a and a‐c domain wall variants coexist (60–90 °C, marked by the gray‐shaded regions in Figure 3a, wherein the domain configurations are largely metastable and heavily influenced by temperature variations. See Video S3 in the Supporting Information. The results here shown are reproducible in subsequent annealing cycles and other BTO samples. Surprisingly, the heating/cooling rate did not appear to significantly influence the trend here observed (Figure S4, Supporting Information) unlike recent work in free‐standing PbTiO_3_.^[^ ^28^ ^]^ This observation suggests that the effect of the heating rate on the domain behavior may be material‐dependent. However, we did observe slight variations in the T_C_ values and the temperature range at which domains coexist across different samples. This is likely due to the lamella dimensions and boundary conditions, such as thickness and potential clamping to the bottom chip, as reported elsewhere.^[^ ^37^, ^40^, ^41^ ^]^

The change in domain configuration, from a‐c to a‐a between RT and 60 °C, is typically not observed in bulk but it resembles similar behavior to that reported in low‐strain BTO thin films^[^ ^42^, ^43^ ^]^ and is comparable to recent observations for BTO lamellae in situ studies.^[^ ^36^, ^37^, ^44^, ^45^, ^46^ ^]^ At this point, it would be tempting to suggest that this domain reconfiguration is due to the material accessing the next low‐temperature phase transition, from tetragonal to orthorhombic,^[^ ^47^ ^]^ allowed perhaps due to the low‐strain regime of the BTO lamellae. This would suggest an almost transitional character for BTO in a free‐standing form, similar to that reported in low‐strain BTO thin films.^[^ ^43^ ^]^ Another plausible explanation would be due to the built‐in electric or stress fields varying at different temperatures. However, this requires further investigation. Nevertheless, the fact that this is not the first time that this behavior has been reported in BTO lamellae, demonstrates that this is not unique to the data here shown and strongly supports that interesting phenomena can be induced in free‐standing ferroelectrics.^[^ ^28^, ^48^ ^]^

An important observation of this study is that the T_C_ here observed is higher and occurs over a wider temperature range (±10 °C) than that of bulk BTO,^[^ ^49^ ^]^ at ≈190 versus 120 °C. To verify the accuracy of the temperature measurement, a LaAlO_3_ (LAO) lamella of similar dimensions was heated under UHV conditions, with identical conditions as the BTO lamella. In this case, the lack of domain contrast was observed near the bulk's T_C_ value^[^ ^50^ ^]^ (Figure S6 and Video S2, Supporting Information), thus confirming the measured T_C_ by in situ STEM for BTO. As an additional test, in situ UHV temperature‐controlled PFM was performed on a sister BTO lamella (Figure S5, Supporting Information) to further investigate the temperature‐induced domain behavior and to rule out beam‐induced effects by STEM imaging.^[^ ^51^ ^]^ Vertical and lateral PFM performed at ≈27 °C confirmed that the domain microstructure is comprised predominantly of a‐c domains. Upon cycling the temperature between ≈27°C and ≈187 °C, a gradual transition between a‐c and a‐a domains was observed, consistent with the in situ STEM observations discussed above. Additionally, a drastic drop in PFM amplitude was observed at the highest temperature of ≈187 °C, indicating a T_C_ range of ≈157–187 °C, which is higher than the bulk and in close agreement with the T_C_ observed by in situ STEM.

Diffuseness on the phase transition resembles what is widely reported for strained ferroelectric thin films,^[^ ^52^, ^53^ ^]^ elucidated to originate from the strong polarization‐strain coupling. Similarly, strained ferroelectric thin films have shown significant shifts in T_C_ when compared to the bulk, for example, a broadening of the T_C_ at ≈176 °C was reported for BTO thin films^[^ ^54^ ^]^ whilst biaxially compressed BTO thin films displayed a T_C_ between 420 and 680 °C.^[^ ^55^ ^]^ Notably, a variety of single crystal BTO nanoparticles have also been reported to show diffuse phase transition.^[^ ^56^, ^57^, ^58^ ^]^ In our case, the sample is a relatively thick free‐standing film and the T_C_ would be expected to be closer to that of the bulk, as reported for capacitor structures using similar single crystal BTO lamellae.^[^ ^59^, ^60^ ^]^ A possible origin for the difference here observed could be the presence of oxygen vacancies (𝑉_o_). Annealing under UHV conditions, even under low temperatures, is known to promote the formation^[^ ^23^ ^]^ and redistribution^[^ ^61^ ^]^ of 𝑉_o_, as well as partially removing atmospheric adsorbates from the sample surface.^[^ ^62^ ^]^ The concentration and distribution of 𝑉_o_ are known to modify the structure via changes in the oxidation states and their coupling with lattice strain.^[^ ^63^, ^64^, ^65^, ^66^ ^]^ 𝑉_o_ are associated with domain pinning,^[^ ^67^ ^]^ accumulation close to 90° domain walls,^[^ ^68^ ^]^ and ferroelectric degradation.^[^ ^69^ ^]^ Importantly, V_o_ are positively charged defects and can significantly affect the electrostatic boundary conditions, hence influencing the screening mechanism. It has been reported that under reducing conditions oxygen deficiency favors BaO evaporation, creating a Ti‐rich surface.^[^ ^70^, ^71^ ^]^ In fact, thermal annealing between UHV and atmospheric conditions in PZT thin films has been shown to induce a reversible effect, between insulating and conducting behavior, demonstrating that surface adsorbates alter the boundary conditions (by varying the amount and distribution of *V*_o_ and the removal of surface adsorbates).^[^ ^72^ ^]^ Whilst the exact origin for the higher and diffuse T_C_ is not clearly known to the authors, we suggest that it can be rationalized by the creation of a defective surface with a highly nonstoichiometric nature, e.g., the creation and redistribution of V_o_, or, forming a surface layer with different chemistry to that inside the “bulk” of the lamellae.^[^ ^61^, ^70^ ^]^ Next, we compare the behavior observed under UHV to that under an oxygen‐rich environment, where a lower concentration of 𝑉_o_ would be expected_._

### 2.4 Oxygen‐Rich Environment

After the UHV cycle, the sample was removed from the TEM, and the full NR was assembled. The first environment tested was N_2_, an inert gas that possesses no charge and was not expected to influence the screening mechanism. Hence, considered a control test before exposing the sample to another environment (reducing or oxidizing). However, the domain behavior observed under N_2_ was unexpectedly different from that under UHV and very similar to that under 20% O_2_/Ar. For this manuscript's brevity, the full data for the N_2_ environment is discussed in Figure S7 (Supporting Information), and shown in Video S3 (Supporting Information). After exposure to N_2_, the sample was directly exposed to an oxygen‐rich environment: 20% O_2_/Ar. In both cases, the NR pressure was 900 mbar, with a flow of 0.3 mL min^−1^, and these conditions were stabilized before annealing. The annealing conditions were identical to that of the UHV cycle: RT – 250 °C – RT at 1°C s^−1^.

The motivation for exposing the ferroelectric surface to an oxygen‐rich environment is twofold: i) to compare the domain behavior to that under UHV, which, whilst commonly used for most in situ TEM experiments, such conditions are not representative of working environments for a ferroelectric, and ii) to lower the population of V_o_ induced by the UHV cycle. The latter is particularly interesting because a fully reversible system would be extremely attractive for chemical switching applications.^[^ ^13^ ^]^

Analogous to the UHV cycle, the domain behavior in the oxygen‐rich environment exhibited repeatability, in a hysteretic fashion for the full annealing cycle (Figure 3d and Video S4). However, there were marked differences. Figure 3e,f displays the STEM images acquired during cooling down, at 80 °C and RT, respectively. At both temperatures, the domain structure is drastically different from that observed under UHV. At 80 °C (Figure 3e), the a‐a domains are confined to the top left part of the sample, while a few a‐c domains can be seen in the bottom part of the field of view. At RT (Figure 3f) the a‐c domains are again the predominant domain variant, but these domains are only present on the right‐hand side of the field of view. The key differences found under the oxygen‐rich environment are: i) the dense domain structure is largely suppressed (decreased), with smaller fractions of both a‐a and a‐c domains (e.g., reducing from ≈0.6 to 0.2 for a‐a domains), ii) each domain variant appears to be confined to defined temperature ranges, with almost no coexistence, and iii) the appearance of a mottling pattern, at the bottom half of the lamella, marked by the arrow in Figure 3e. A plausible explanation for this mottling pattern is the formation of gallium oxide at the surface. It is well known that the FIB process causes gallium implantation, which results in an amorphous layer on the lamella surface. The bottom of the lamellae is more prone to damage from re‐sputtered material from the trench sidewalls during FIB milling and therefore can be expected to contain a higher gallium concentration. However, EDX analysis revealed a very low gallium content in the bottom part of the lamellae and even lower in the middle area (Figures S8 and S9, Supporting Information). Furthermore, the mottling was not observed on the LAO sample (Figure S6, Supporting Information) even when all samples were prepared and analyzed under similar conditions. In fact, for both samples, the gallium concentration is relatively low and was found to decrease after the UHV cycle (see Supporting Information). Another possible source for mottling is the creation of another surface species. The surface of BaTiO_3_ has been characterized sometimes as a Ti‐O^[^ ^71^ ^]^ and BaO rich depending on the atmosphere. Spasojevic et. al. identified BaO_2_ formation on bulk BTO in an XPS study during heating in an O_2_ atmosphere.^[^ ^23^ ^]^ However, additional investigations are required to fully identify the surface chemistry.

**Figure 4:**
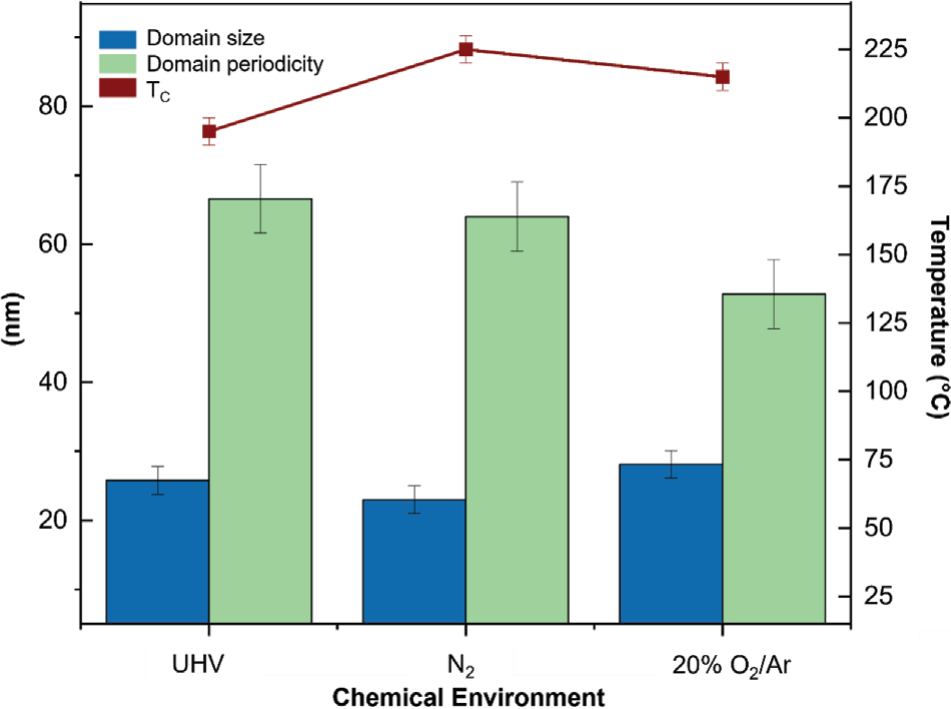
The effect of the chemical environment on domain size and periodicity measured at 90 °C and *T*_C_. The domain size is measured from wall to wall of the same domain, and domain periodicity is measured from the first wall of one domain to the first wall of the neighboring domain; both measured directly from intensity profiles across the domains on HAADF images.

Additionally, it was observed that both the oxygen and nitrogen environments affect the T_C_, domain size, and periodicity_._ **Figure** **4** displays the changes in all environments (UHV, N_2_, and 20% O_2_/Ar) at 90 °C, as this is the temperature at which more domains are observed under the N_2_ and 20% O_2_/Ar environments. The domain size exhibits no evident trend, slightly increasing for the oxygen‐rich environment. On the other hand, the domain periodicity continuously decreases as the sample is exposed to different environments, with the lowest value measured under the oxygen environment. It should be noted that the domain size is measured from wall to wall of the same domain, and domain periodicity is measured from the first wall of one domain to the first wall of the neighboring domain; both measured directly from intensity profiles across domains on HAADF images. The *T*_C_ in all three environments is diffused and much higher than that of the bulk T_C_ (≈120 °C), and that reported in previous BTO lamellae work.^[^ ^59^, ^60^ ^]^ The lowest *T*_C_ is observed for UHV (≈195 °C), followed by oxygen (≈215 °C) and nitrogen (≈225 °C).

As previously mentioned, the T_C_ observed in this work significantly differs from the *T*_C_ reported in previous BTO lamellae work.^[^ ^59^, ^60^ ^]^ However, there are some important differences to be noted.Firstly, the lamella in this work has only a thin top electrode (Pt), unlike the work reported by Chang et al.^[^ ^59^ ^]^ It is well known that electrodes alter the boundary conditions and thus induce different screening phenomena. Additionally, our samples were annealed and observed in situ, under subsequent environments with no exposure to ambient conditions between imaging. The authors attribute the changes observed in the N_2_ and O_2_ environments, particularly the apparent domain suppression, to two likely phenomena: a decrease of V_o_ and a change in surface species. The surface species could form a thin layer that differs from the depth of the sample,^[^ ^73^ ^]^ similar to a dead layer on the surface, which would alter the screening mechanism and prevent the reduction of oxygen vacancies. We propose that the domain suppression observed in the oxygen environment is indicative of the material trying to reach a monodomain state, similar to previous studies on PbTiO_3_ thin films.^[^ ^13^ ^]^ There are several potential reasons for this monodomain state not to be realized. Although there is strong evidence that significant surface compensation is achieved by ionic adsorption for relatively thick films,^[^ ^74^, ^75^, ^76^ ^]^ the lamella thickness might offer different screening mechanisms to that of an epitaxial thin film. The lamella contains two free surfaces, whereas a thin film without a top electrode only contains one. Thus, negatively charged O_2_‐ ions can accumulate at both free surfaces, where the top surface could be more exposed to the gas flow and would create surface layers with uneven stoichiometry that could result in a full preferential polarization orientation not being achieved. It could also be possible that a complete monodomain state is not viable in this configuration. In addition to polarization, BTO requires the spontaneous strain in the system to be compensated when cooling through T_C_, via the formation of 90° ferroelastic domains. Previous work has modeled the effect that the FIB‐induced surface damage has on the domain configuration, where it was found that the driving stress for the formation of periodic 90° domains is provided by an encapsulating surface layer.^[^ ^77^ ^]^ Overall, it should not be ruled out that some of the behaviors here observed are intrinsic to free‐standing ferroelectrics with boundary conditions that diverge from the typical two metal electrodes or thin‐film design, and that the surface ionic and electronic screening would become comparable to the bulk‐free energy of the ferroelectric.

One important aspect to consider is the effect of the sample's history on modifying the screening mechanisms. Interestingly, it was observed that if the sample is directly cycled between UHV, N_2_, and O_2_ environments, without being exposed to air (e.g., kept inside the TEM), the domain behavior could be replicated. However, this reproducibility is lost once the sample is removed from the TEM and exposed to ambient conditions. In this case, high‐temperature annealing can be used to induce the initially observed domain behavior,^[^ ^40^ ^]^ strongly indicating the effect of adsorbates from the environment.

To further investigate the drastic change in domain behavior between environments, the same experiment was conducted on a purely ferroelastic system (a free‐standing LaAlO_3_ lamella), with no polarization component. It was found that the resulting domain structure at RT does not change between the UHV and the nitrogen/oxygen‐rich environments, and the domain structure is not suppressed in any way (Figure S6, Supporting Information). Thus, suggesting that the change observed between the domain behavior in the different environments in free‐standing BTO lamella is strongly related to the exchange between polarization and surface adsorbates.

### 2.5 Pressure Effects

In addition to the effect that the chemical environment can have on the domain behavior, there are conflicting reports about the effect that pressure has on ferroelectrics. In general, it is acknowledged that ferroelectricity is suppressed with increasing hydrostatic pressure, because it increases the short‐range repulsions which favor the nonpolar phase, more rapidly than long‐range interactions that prefer the ferroelectric phase.^[^ ^78^ ^]^ For example, in bulk SrTiO_3_, 66 kbar has been found to be a critical pressure for ferroelectric instability.^[^ ^79^ ^]^ Contrarily, in nitride perovskites, moderate hydrostatic pressures have been found to stabilize the ferroelectric phase, which is only metastable under ambient conditions.^[^ ^80^ ^]^

The experimental design of the DENSsolutions Climate holder allows for a small pressure to be achieved (max ≈ 1 bar). Additionally, there is a natural compromise to achieve between flow, temperature, and pressure to avoid the windows of the nanoreactor (NR) from breaking. Nevertheless, it is possible to vary the pressure while keeping the gas flow rate (0.3 mL min^−1^) and temperature constant. It is worth mentioning that some degree of bulging can be expected from the silicon nitride window as the temperature increases.^[^ ^81^ ^]^ This deflection, in the Z‐direction, is more significant at higher temperatures (>300 °C) and typically observed by a drastic change in focus, which was not observed here. Therefore, this effect is expected to be minimal for the temperature here chosen (80 °C).

**Figure 5:**
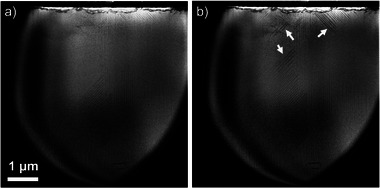
Pressure effect on domain microstructure, under O_2_/Ar at 80 °C and 0.3 mL min^−1^. a) 900 and b) 700 mbar . White arrows in (b) indicate positions where domain walls have nucleated after the pressure was reduced.

Under 20% O2/Ar, it was found that reducing the NR pressure by 200 mbar at a constant flow rate and temperature favors the nucleation of new domain walls, as shown in **Figure** **5**. This can be rationalized by addressing the concentration of the O_2_ molecules present in the NR, which is higher at 900 mbar (Figure 5a) than at 700 mbar (Figure 5b). An important factor mentioned in previous reports is the partial pressure of oxygen (*p*O_2_). For example, the polarization orientation can be inverted in ultra‐thin PbTiO_3_ by changing between low and high *p*O_2_.^[^ ^13^, ^62^ ^]^ Furthermore, the temperature dependence of the polarization is a strong function of the external *p*O_2_. At intermediate values, the *T*_C_ in the film is strongly suppressed.^[^ ^1^ ^]^ Therefore, it could be that the increased pressure causes more adsorbates to bind to the surfaces of the lamella, changing the screening conditions and successfully compensating the depolarizing fields.

However, the introduction of the inert N_2_ atmosphere was also found to change the domain structure significantly. Thus, it could be the flow or pressure of N_2_ changing the current adsorbate structure (H_2_O, OH─CO_2_, etc.) on the surface from the start, which would change the screening conditions and hence the preferred polarization orientation for subsequent environments. In support of this argument, there is the work of Segura et al.which revealed a gradual incorporation of positive charges onto the surface of a PZT thin film during low humidity (N_2_) experiments,^[^ ^82^ ^]^ and considered it unlikely that such charges came from new adsorbates in the environment, but this was not fully rationalized.

In this work, it is difficult to ascertain whether it is the presence of adsorbates, the creation, and diffusion of *V*_o_, or the pressure and flow in the NR, that causes the significant changes to the domain microstructure between UHV and nitrogen/oxygen enviroments. Potentially, a limiting factor is the FIB processing, which creates an amorphous gallium‐implanted surface layer that encapsulates the material of interest. This would limit the ability of the charge to be compensated by adsorbates, which is purely a surface phenomenon. This is an important aspect to address in future experiments, because the full potential to study the ferroelectric surface by this technique may be limited by the sample preparation.

Finally, for completeness, the temporal effect was also investigated and in general, it was observed that the domain structure did not change as a function of time, see Supporting Information.

## 3 Conclusion and Outlook

In this study, we employed in situ STEM gas and heating to investigate the impact of different chemical environments on ferroelectric‐ferroelastic domains in free‐standing films of barium titanate (BTO). By cycling the sample from RT to 250 °C, past *T*_C_, we examined the behavior of the domains under three different environments: ultrahigh vacuum (UHV), nitrogen (N_2_), and oxygen‐rich (O_2_/Ar) conditions. Our results revealed distinct domain behavior under each environment. Specifically, the UHV environment promoted a dense metastable configuration of competing a‐c and a‐a domain variants, which was corroborated by in situ heating PFM studies. In contrast, both N_2_ and oxygen/argon (O_2_/Ar) environments largely suppressed the domain structure immediately after gas flow, and the oxygen environment created a mottled feature indicative of a surface reaction. Additionally, we established an automated pathway to statistically disentangle and detect domain structure transformations using deep autoencoders, providing a pathway toward real‐time analysis. Our results emphasize the importance of considering the chemical environment when performing in situ experiments on ferroelectric materials, particularly under UHV conditions. Additionally, we suggest that the pressure effect should be further investigated as a route to understand and control the interaction between the ferroelectric surfaces and the environments’ chemical species. To the authors' knowledge, this is the first time that such experimental techniques (in situ gas and heating STEM) have been applied to study environmental effects on domain configurations in free‐standing ferroelectric films, which have recently emerged as an exciting platform as they exhibit interesting effects that differ from the bulk.^[^ ^26^, ^27^, ^28^, ^29^ ^]^ Hence, we expect that this study would motivate further experimental and theoretical in situ gas STEM studies on ferroelectrics.

## 4 Experimental Section

Lamellae of ≈150 nm thickness were prepared from BaTiO_3_ (BTO) and LaAlO_3_ (LAO) single crystals using a dual‐beam FIB TESCAN LYRA3 and transferred to DENSsolutions climate MEMs chip via ex situ lift‐out technique,^[^ ^41^, ^83^ ^]^ with the lamellae positioned as shown in Figure 1 and Figure S1 (Supporting Information). The BTO single crystal was sputtered with a 225 nm thick Pt layer to help protect the surface during FIB milling. Prior to in situ heating and under vacuum, the quality of the lamella was imaged under static conditions to check for preferential thinning, thickness gradients, or any bending/ curtaining that could lead to strain gradients, similar to those previously reported.^[^ ^41^ ^]^ A sister sample was prepared for piezoresponse force microscopy (PFM) with a similar procedure as for the STEM samples, but with a thickness of ≈300 nm on a platinized substrate; and acid washed with HCl to remove the gallium oxide layer expected to form at the surface from the FIB sample preparation.^[^ ^84^, ^85^ ^]^

Scanning transmission electron microscopy (STEM) images were acquired simultaneously on high‐angle annular dark‐field STEM imaging (HAADF) and bright‐field STEM (BF‐STEM) detectors, to image 90° ferroelectric‐ferroelastic domains, as the contrast can arise from differences in the Bragg condition (e.g., lattice distortion from one domain to another); 180° domains are observable to a lesser extent due to the contrast arising due to the possible failure of Friedel's law.^[^ ^86^ ^]^ STEM was performed on an FEI TALOS F200 G2 at 200 kV. Low‐current imaging (≈50 pA) was used to prevent electron beam‐mediated domain nucleation.^[^ ^51^ ^]^ The temperature was gradually changed using a ramp of 1°C s^−1^, and an image was acquired every 5 °C with a dwell time of 20 µs. In situ heating PFM under ultra‐high vacuum (UHV) conditions was performed on a Scienta Omicron VT‐AFM system, between ≈27 °C and ≈187 °C with vertical and lateral PFM scans taken at 7‐predetermined temperatures, guided by the STEM in situ heating data. Temperature stills from this heat cycle are shown in Supporting Information. The temperature of the stage was controlled via a resistively heated cold finger and was monitored with a gilded copper thermalization block located in contact with the sample holder. Under these conditions, it should be noted that the temperature at the sample surface is estimated to be ≈5–10 °C lower than is recorded on the temperature sensor. During in situ experiments, the TEM column operated at a vacuum level of 10^−8^ mbar and the base vacuum of the UHV PFM system was ≈10^−11^ mbar.

## Conflict of Interest

The authors declare no conflict of interest.

## Supporting information

Supporting Information

Supplemental Video 1

Supplemental Video 2

Supplemental Video 3

Supplemental Video 4

Supplemental Video 5

## Data Availability

The data that support the findings of this study are available from the corresponding author upon reasonable request.
