# Supplementary material for: The Effect of Chemical Environment and Temperature on the Domain Structure of Free‐Standing BaTiO3 via In Situ STEM
Source: Adv Sci (Weinh). 2023 Aug 21;10(29):2303028. doi: 10.1002/advs.202303028 (PMC10582436; doi:10.1002/advs.202303028)
Supplement: Supplementary file 1 — Supporting Information [file ADVS-10-2303028-s005.pdf]

ADVANCED SCIENCE
Open Access

Supporting Information

for *Adv. Sci.*, DOI 10.1002/advs.202303028

The Effect of Chemical Environment and Temperature on the Domain Structure of Free-Standing $BaTiO_3$ via In Situ STEM

Tamsin O'Reilly, Kristina M. Holsgrove, Xinqiao Zhang, John J. R. Scott, Iaro Gaponenko, Praveen Kumar, Joshua Agar, Patrycja Paruch and Miryam Arredondo**

Supplementary Information for:

The effect of chemical environment and temperature on the domain structure of free-standing $BaTiO_3$ via in-situ STEM

Tamsin O'Reilly[1,2], Kristina M. Holsgrove[1], Xinqiao Zhang[3], John Scott[1], Iaro Gaponenko[4], Praveen Kumar[1,5], Joshua Agar[3], Patrycja Paruch[4] and Miryam Arredondo*[1]*

1. *School of Mathematics and Physics, Queen's University Belfast, BT7 1NN, United Kingdom 2. University of Glasgow, United Kingdom 3. Department of Mechanical Engineering and Mechanics, Drexel University, PA, United States 4. DQMP, University of Geneva, 1211 Geneva, Switzerland. 5. Shared Instrumentation Facility, Colorado School of Mines, Golden, CO, United States*

E-mail: toreilly03@qub.ac.uk, m.arredondo@qub.ac.uk

1. In-situ set up.

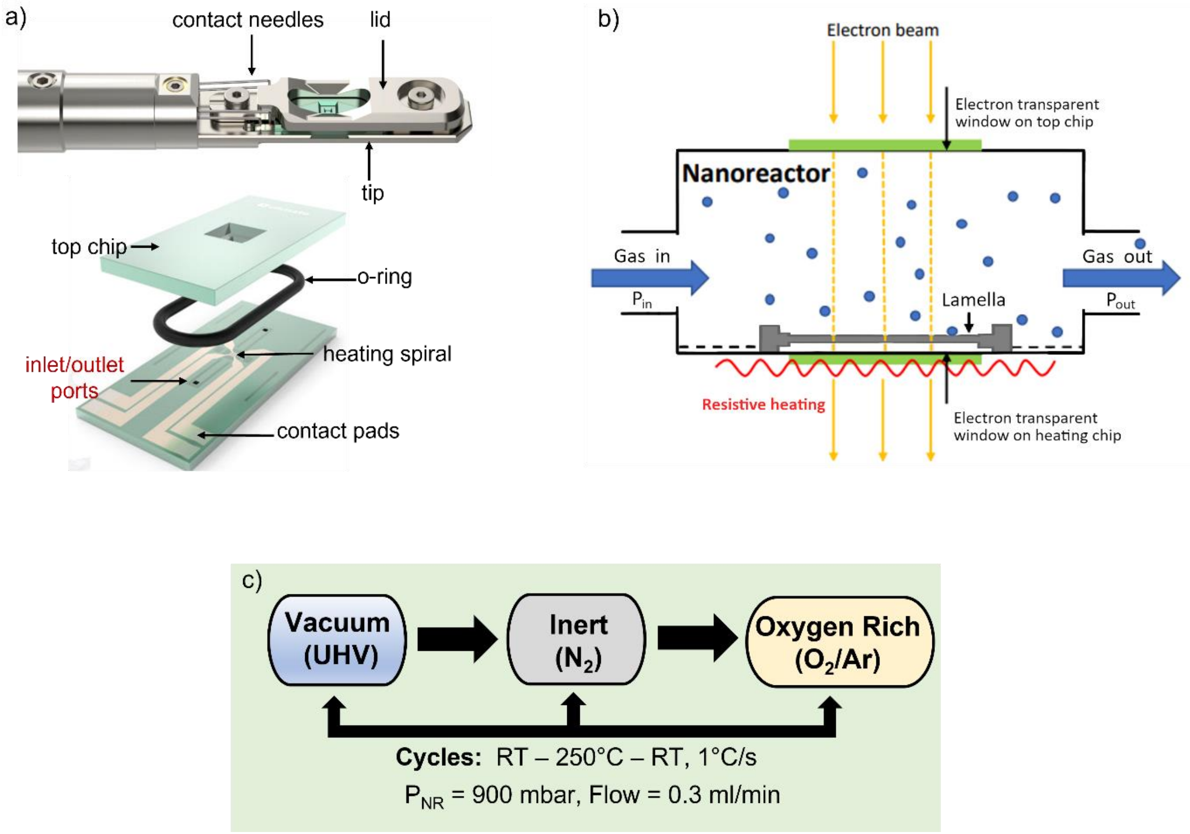

Figure S1. Overview of the in-situ Climate DENSsolutions holder and experimental conditions. a) Holder configuration and schematic of the top and bottom chip which form the full nanoreactor (NR), adapted from,[1] b) Schematic representation of the NR fully

assembled, indicating the lamella position and electron beam direction, not to scale, and c) overview of the experimental conditions and changes in atmosphere.

2. **Bright-Field Image Segmentation of BTO Domain Response to Environmental Stimulus.**

2.1. Constructing Domain Representation from Embedding Layer

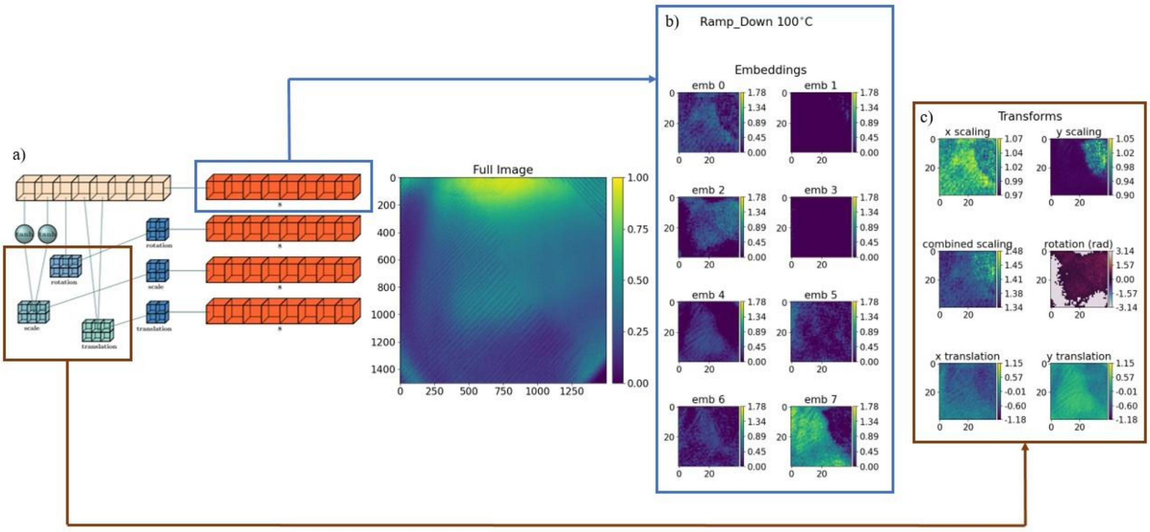

Fig. S2. Examples of embedding from ramp down at 100°C. a) Latent space. b) Embeddings constructed from reshaping the first 8 dense points from the full dataset. c) Transforms taken from the transformation matrices generated from the first 5 dense points.

2.2. Relative Domain Area Calculations

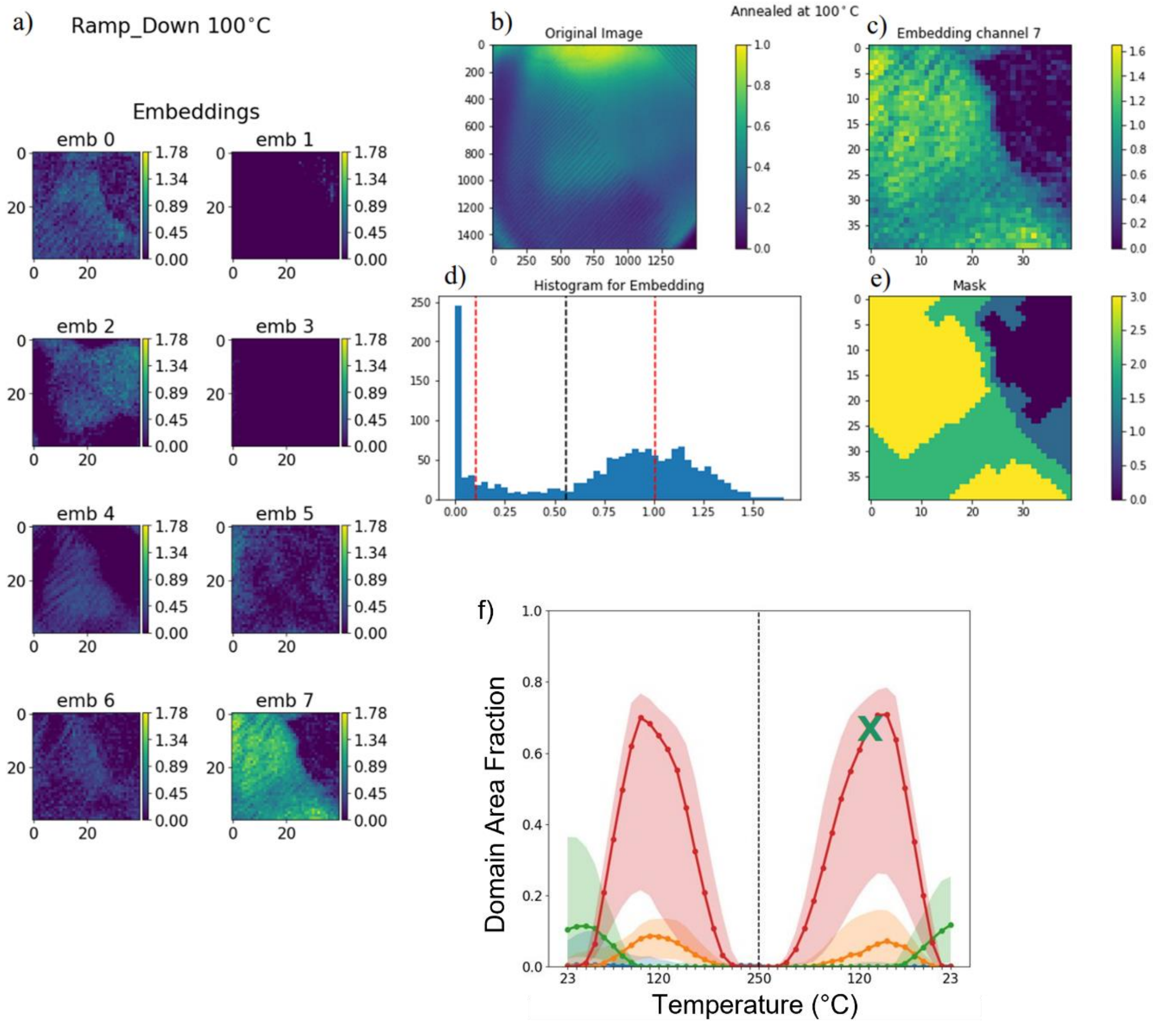

Fig. S3. a) Reshaped embedding images shown. b) The original image of the annealed sample on the 100°C ramp down is shown. c) Embedding channel 7 represents the a-a domain. d) the Otsu Threshold is shown by the black vertical line in histogram, and the uncertainties by the red lines. e) Generated mask is shown by the green area in the mask, the lower uncertainty is shown by yellow, and the upper uncertainty shown by blue. f) The relative areas for each environment and domains are shown by solid lines, while the shading represents the uncertainties. The green X marks the domain and temperature corresponding to the mask example. The videos for each environment can be found as separate files.

3. Heading rate effect.

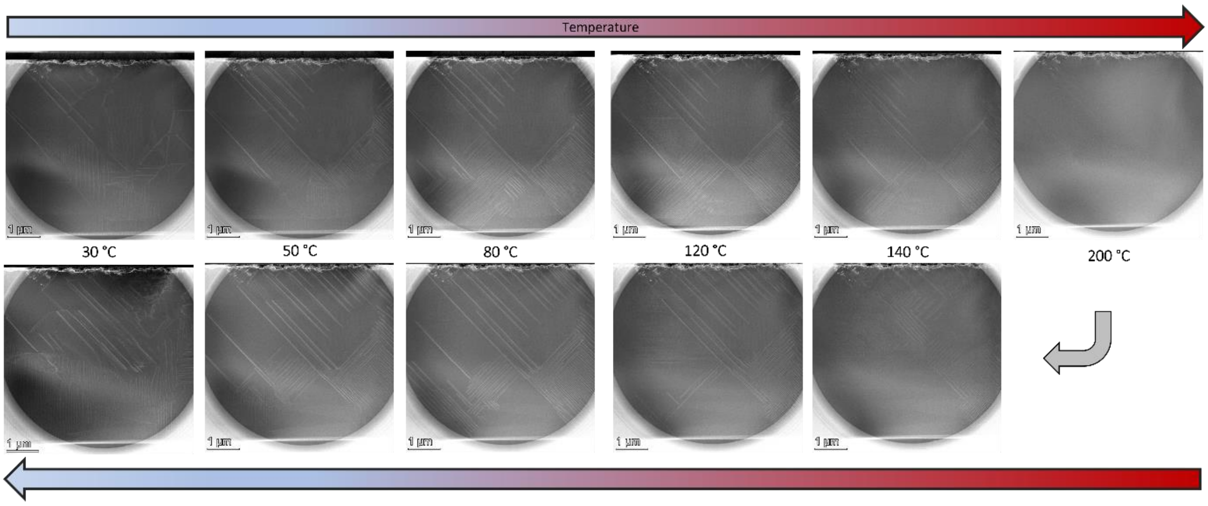

Fig. S4. HAADF- STEM images of a sister BTO lamella with a ramp rate of 5°C/sec, under UHV, from RT to 200°C and back to RT. This ramp is five times faster than the ramp rate used in the data set of the main manuscript (1°C/sec). Here a mixture of a-a and a-c domains can be observed at temperatures as low as 30°C, and these domains co-exist at temperatures as high as 140°C. However, a similar trend to that described in the main manuscript is observed. This is that the predominant domain variant at low temperatures is the a-c domains, and the a-a domains are the dominant domains at high temperatures. The T_C is also diffused at ~ 220°C, following the overall behavior described in Fig. 2.

4. In-situ Heating PFM

To confirm the TEM observations, in-situ UHV temperature controlled PFM was performed on a sister BTO lamella with a similar procedure as for the TEM samples but with a thickness of ~300 nm on a platinized substrate; and acid washed with HCl to remove the gallium oxide layer expected to form at the surface from the FIB sample preparation.[2, 3] The UHV PFM was performed with Dual Frequency Resonance Tracking (also known as the proprietary DART-PFM technique on the Asylum Research AFMs), using platinum silicide coated cantilevers - Bruker SCM-PTSI, 75kHz, 2.8N/m. The frequencies employed were at the vertical contact resonance for vertical PFM, and at the lateral contact resonance with lateral PFM - around 400kHz and 800kHz respectively. The PFM excitation voltage was set to 2V, but the deflection force was not calibrated (but the deflection setpoint was set to usual contact mode AFM conditions in both cases, resulting in forces in the range of 100-300 nN).

The vertical and lateral phase and amplitude PFM micrographs are shown in Figure S5. As described in the main text, the observed T_C is consistent with the in-situ TEM measurements.

Additionally, no drastic domain structure changes are observed during the temperature ramp up; and when the temperature is lowered from past T_C, a domain configuration similar to the one during ramp up is observed – hinting at the presence of defects or pinning sites that will reproduce the observed polarization structures.

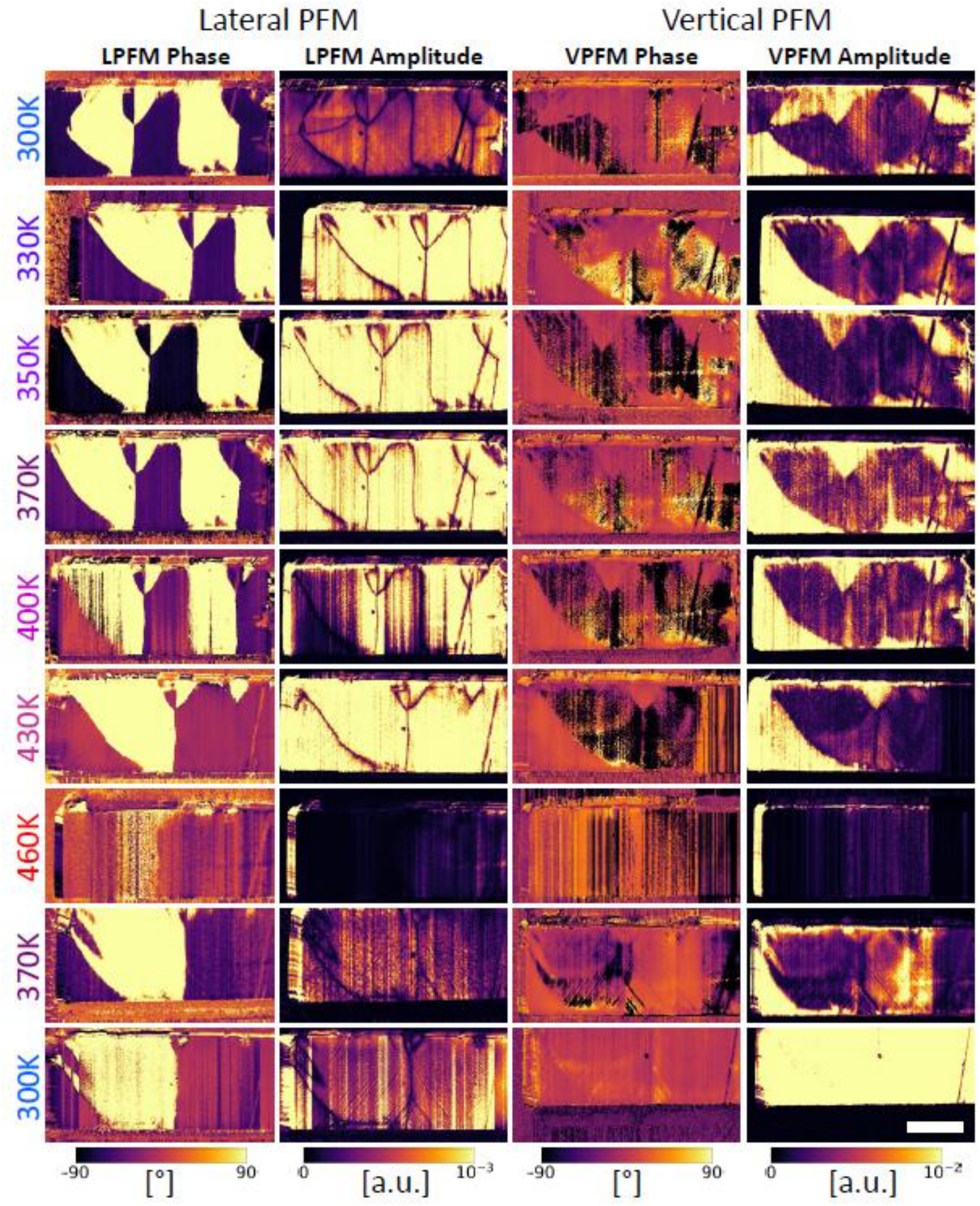

Figure S5. In-situ UHV temperature controlled piezoresponse force microscopy micrographs. Each row is a set of vertical and lateral PFM measurements at a different temperature, with temperatures ramped up from ~26°C (300K) to ~186°C (460K), and then lowered down to ~26°C to observe domain structure evolution and recovery. During the temperature ramp up, the domain structure gradually evolves, but remains comparable. The loss of vertical and lateral PFM amplitude at 460K clearly indicates that the T_C has been reached. As the temperature is lowered, the previous domain

structure is partially recovered, indicating the presence of pinning sites or defects favoring specific domain configurations. The scale bar in white represents a length of 1 micrometer and is identical to all images. Due to the open loop nature of the microscope's scanner, distortions are visible throughout the measurement series.

5. $LaAlO_3$ (LAO) free-standing thin film: In-situ observations under different environments.

Figure S5 shows HAADF-STEM images at RT for a LAO lamellae cycled between different environments: UHV, nitrogen and 20% O_2/Ar at 0.28ml/min and 950 mbar. The heating rate was 2°C/min from RT to 800°C and back to RT. LAO has a T_C much higher than BTO, ranging from ~465°C to 575°C.[4-6] The lamellae here investigated are from the same bulk sample previously investigated by optical in-situ heating, where the T_C was found to be ~575°C.[7]

At around 600±5°C there is no clear domain structure visible by either HAADF-STEM or DF-STEM (not shown here), indicating a transformation from the ferroelastic to the paraelastic phase (video V2). The as grown sample shows no domain structure at RT, prior to annealing under UHV, however a rich domain structure develops after, possibly due to the new elastic boundary conditions. Under the nitrogen and oxygen this domain structure does not change significantly. This would indicate that, as expected, the presence of surface adsorbates does not affect the domain formation. If anything, it could be expected that oxygen vacancies would play a more significant role. Importantly, the observed Tc remains ~545±5°C for all environments and some degree of diffuseness is observed, although this is not as marked as for the BTO lamellae.

EDX analysis from the bottom of the lamellae indicate the changes for gallium, oxygen, and nitrogen, before and after annealing under UHV. In general, the gallium content is in average under 10% and this decreases to ~ 5% after annealing. It should be noted that the end of the lamellae would be slightly thicker due to the FIB processing. In contrast the nitrogen and oxygen content do not appear to change much with annealing under UHV and remain ~16% and 24% respectively. It should be noted that measuring nitrogen and oxygen content by EDX is challenging and should be taken with caution. Thus, these measurements should be taken more like a trend, given that these were acquired over the same area with the same acquisition conditions. Finally, changing the pressure by 200 mbar under nitrogen (N_2) did not modify the domain structure.

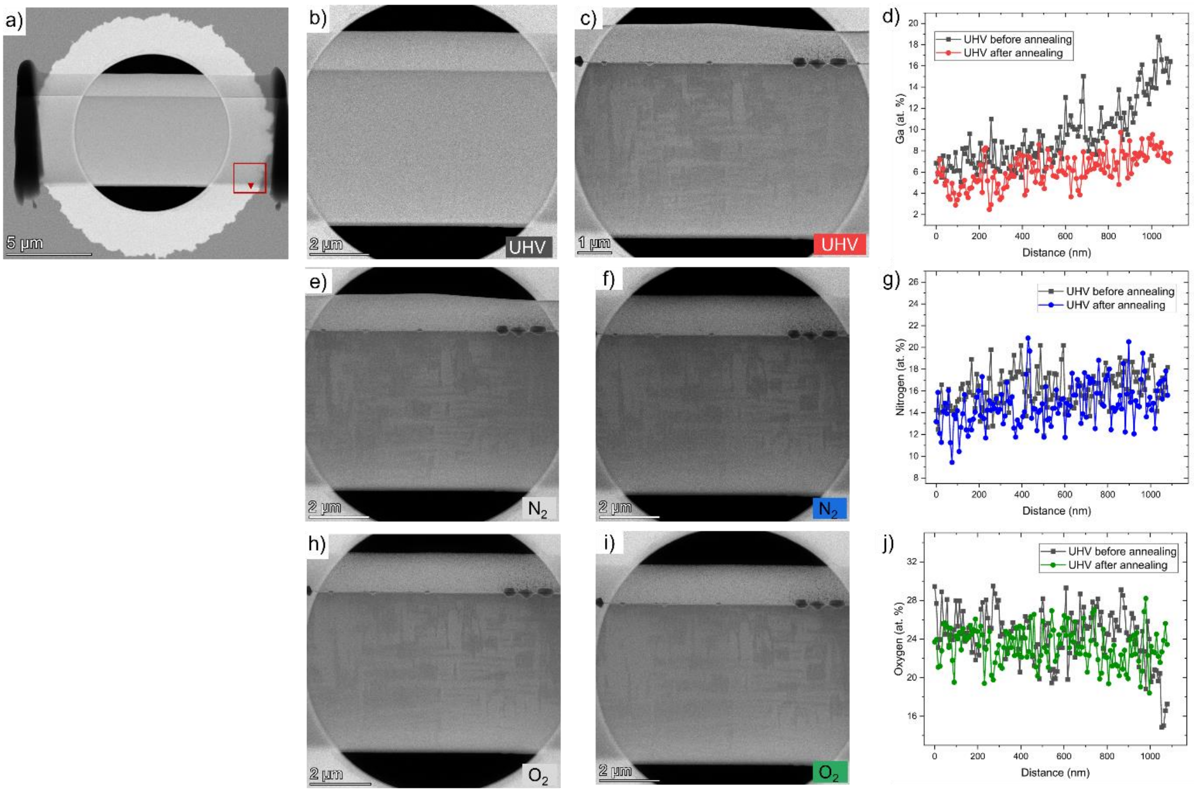

Fig. S6. HAADF - STEM images of a LAO lamellae under different environments, after annealing at 2°C/min from RT to 800°C and back to RT. a) Sample overview at RT, the red box indicates the area from which EDX was acquired. b) and c) are the overviews at RT for before and after annealing under UHV, respectively. e) and f) are the overviews at RT for before and after annealing under nitrogen, respectively. h) and i) are the overviews at RT for before and after annealing under oxygen, respectively. d), g) and j) are the EDX atomic % profiles for Ga, nitrogen, and oxygen, respectively acquired before and after annealing under UHV.

6. In-situ heating under an inert environment: N_2

The first chemical environment that was investigated right after vacuum, was nitrogen (N_2), an inert gas, a non-polar molecular which is not expected to strongly interact with the ferroelectric surface. Based on the latter, it was assumed that N_2 flow would have very little effect on the domain microstructure or the in-situ behaviour of the lamella. Therefore, this would act as a good control experiment before introducing the oxygen (O_2) environment. However, it should be considered that for this first transition between UHV to gas, the sample was taken out of the TEM to assemble the full NR, and in this process is likely that some additional species could have been adsorbed on the surface of the lamella. It is known that the exposure of a ferroelectric surface to ambient conditions leads to the incorporation of surface species such as molecular

water, hydroxyl (OH) groups and different carbon-related species (CO_x). As previously stated, the adsorption of chemical species can greatly modify the surface chemistry[8, 9] and in turn, change the depolarization field and associated electrostatic energies,[10-13] with surface ions significantly affecting the polarization (ionic screening) and temperature behavior in ferroelectrics.[14] Thus, we now experimentally evaluate how ferroelectric domains respond to different subsequent environments.

Surprisingly, the domain microstructure significantly changed after the flow of N_2 at RT, see Fig. S3. This flow of N_2 resulted in a suppression of the a-c domains variant and a change in the 180° sawtooth domain configuration, Figs. S3b and S3c., with the area fraction reducing from ~ 0.7 to ~ 0.3. During *in situ* heating, the transition from a-c to a-a domains still occurred, albeit at slightly different temperatures than in the UHV environment. The two main domain variants coexisted over a smaller temperature range (80°C – 100°C) in N_2 flow, than in vacuum (60°C – 90°C), with the domains occupying markedly different portions of the lamella, with little interaction between them (video V3). Moreover, T_C was found to be ~ 225°C, higher than in vacuum.

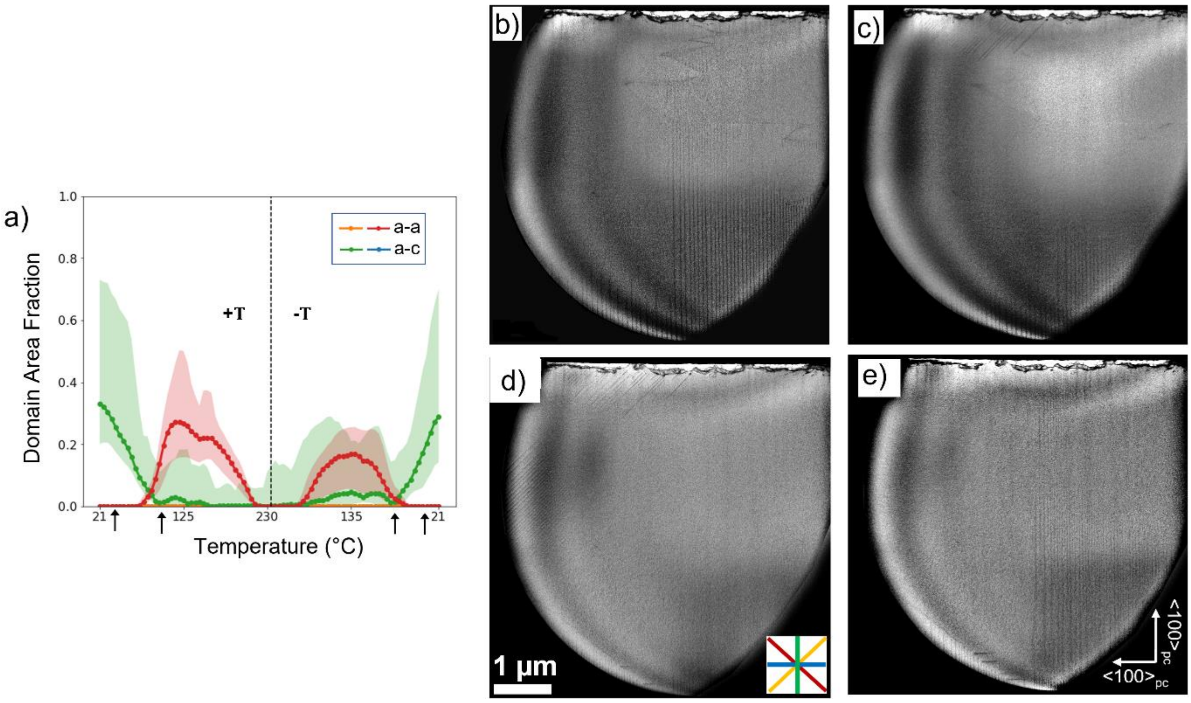

Figure S7. Domain microstructure during heat cycling, at 1°C/sec, under nitrogen (900 mbar, 0.3 ml/min). a) is the relative domain area fraction observed for the full heating cycle, RT - 250°C – RT, where +T and -T indicate heating and cooling, respectively. BF STEM images displaying the domain structure present at b) 25°C, c) 80 °C, d) 80 °C and e) 25 °C, respectively. These reference temperatures are marked by black arrows in a). The inset in d) shows the orientation of the domain

variants as color coded in a). The black circular contour around the sample in the STEM images indicates the bottom and top chips from the NR.

Some points are discussed below, regarding possible reasons for the change in domain structure upon N_2 flow at RT:

a. A change in the screening conditions at the ferroelectric surface: N_2 is a small molecule, that would likely be physiosorbed onto the surface, aided by the flow and pressure within the small NR. However, N_2 is an inert gas, with no external charge available to compensate the depolarising field, so even if adsorbed, it is not expected to change the screening conditions.

b. Point defects: It is also unlikely that flowing N_2 at RT would affect the number of oxygen vacancies (Vo) or other point defects in the material, considering that previous heating cycles have been performed to 250°C in UHV, where the promotion of Vo is expected.

c. It could be argued that the adsorption of N_2 is potentially changing the current adsorbate structure (H_2O, OH-, CO_2 etc.), present on the BTO's surface due to the exposure to ambient conditions during the NR assembly. Such adsorbates screen the polarisation charge from the surface, decreasing the depolarisation energy as well the surface energy.[15] A change in the adsorbate structure would therefore change the equilibrium domain structure. Dohnalek et al. studied the physisorption of N_2 on fully oxidised TiO_2 (110) surfaces using beam reflection and temperature-programmed desorption (TPD) techniques. At high coverages of N_2, it was found that most Ti^{4+} sites became occupied and the close proximity of the adsorbates to their neighbouring positions resulted in strong repulsion.[16] It is not unfeasible that the N_2 flow could also promote adsorbate diffusion on the surface, which would change the level of screening available. However, the adsorption of molecules on ferroelectric surfaces is not a random process, but somewhat dependent on the dipole orientation. It is probable that the adsorbates are already in an ordered fashion and presumably in low energy positions. Furthermore, when the lamella is being imaged at low temperature, it is unlikely that the adsorbates are excited with kinetic energies larger than the diffusion barrier.[17] One argument that supports that N_2 flow is affecting the adsorbates, is that the RT configuration changes after the lamella is heat cycled from 21°C to 250°C and back to 21°C. For example, the 180° sawtooth domain disappears following the heat cycle in N_2, See Figs. S3b and c. Further to this, a previous literature report revealed a gradual incorporation of positive charges onto the surface of a PZT thin film during low humidity (N_2) experiments.[18] The

authors considered it unlikely that such charges came from new adsorbates in the environment, but the generation of the charge was not fully explored.

d. Electron transparent window bulging induced by the gas flow: The Si_3N_4 thin film covering the bottom MEMs chip has bulged due to the sudden flow of gas, causing a change in height of the specimen. If the film has bulged unevenly, it will affect the visualisation of the domains in different areas of the lamella and it would induce inhomogeneous strain across the sample. However, there was no obvious signs of bulging before and after the gas flow of gas (no change in focus across the lamella).

e. The pressure within the NR: In UHV conditions, the pressure in the TEM is ~$6x10^{-8}$ mbar in comparison to the near atmospheric pressure in the NR during N_2 flow, ~ 900 mbar. Initial studies on pressure effects suggest that the domain configurations are somehow affected by increasing pressure.

More than one of the above points seem likely, or even a combination of these. Unfortunately, at this point the exact reason as to why the domain pattern changes with the flow of N_2, even at temperatures as low as 25°C is not clear. Following the introduction of 20% O_2/Ar into the NR there were no obvious changes to the domain configuration from that in N_2, which was unexpected as it was thought that the oxygen rich environment will induce a drastic change in the domain behaviour.

7. BTO: EDX Analysis

The gallium and oxygen content were measured for several samples before and after annealing under UHV, as shown in Fig. 8. In general, the gallium content is always low, <5 at. %, increasing at the bottom of the lamellae due to FIB milling making effects. And the oxygen content remains constant before and after annealing.

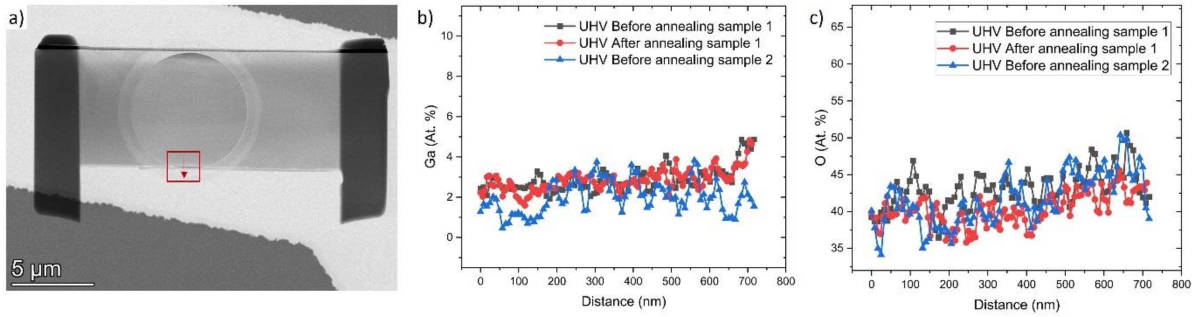

Figure S8. EDX analysis on the surface of the BTO lamellae.

a) HAADF STEM image of the lamella before heating from RT to 250°C at 1°C/sec under UHV, the red box and arrow indicate the area from which EDX was acquired. b) and c) are the EDX atomic %

profiles for gallium and oxygen, respectively acquired before and after annealing under UHV for 2 samples.

The in-situ heating in a 20% O_2/Ar environment caused the formation of a surface species on the lamella at ~ 190°C, apparent in the form of specks or mottling, which was initially attributed to the formation of gallium oxide. In an attempt to identify the surface species of this mottling, EDX analysis was performed following the in-situ gas experiments, described in the main manuscript. This revealed no significant increase in the gallium concentration in regions that exhibited the mottling effect and the atomic fraction of gallium remained <1% across the whole lamella. Another possibility for this mottling effect could be the formation of barium peroxide. However, this would not be identifiable by EDX analysis and would require further exploration using EELS or XPS to confirm the surface chemical species here being formed. It should also be noted that the content of nitrogen is kept low across the lamellae surface, which would indicate that the surface has not adsorbed significant amounts of nitrogen, being the other chemical species that the sample was exposed to, although measuring the nitrogen content by EDX is challenging and should be taken with caution.

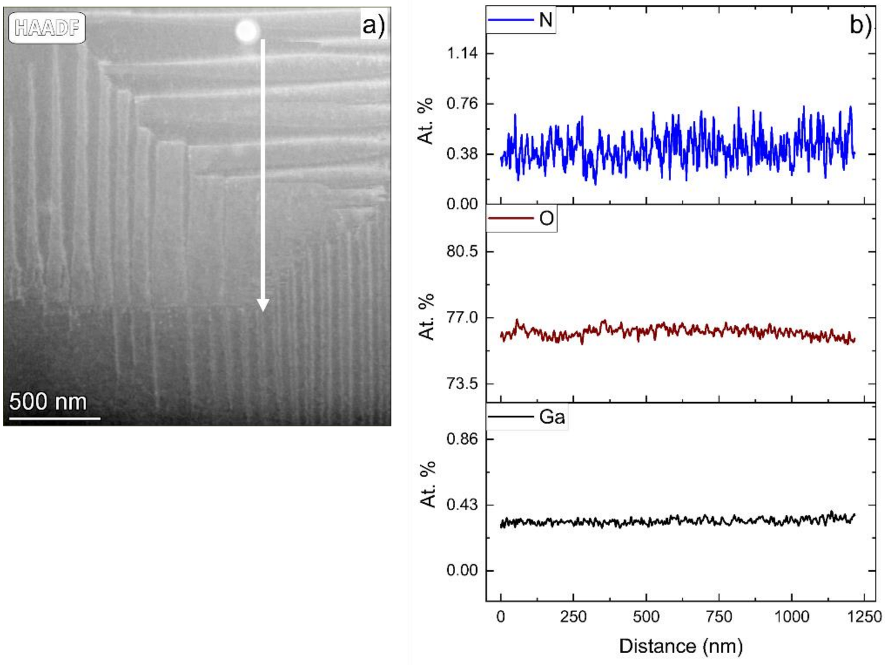

Figure S9. EDX analysis on the surface of the BTO lamellae.

a) HAADF STEM image of the lamella after the oxygen environment. The white arrow indicates the area from which the elemental analysis was acquired. (b) Atomic fraction percentage of oxygen (O), nitrogen (N), and gallium (Ga).

8. Temporal effects

Hydrocarbons, represent a common surface contamination source which profoundly impacts surface composition and the level of surface contamination even under UHV conditions has been reported to change over time.[19] The prolonged exposure of a ferroelectric surface to ambient conditions can lead to an increment of carbon, carbonates and other oxygen species on the surface.[20] It is elucidated that extended exposure to a sealed environment could cause the migration of adsorbates and defects to their equilibrium positions, which would effectively ‘imprint’ the domain microstructure by pinning the domain walls.[21, 22] Such pinning sites cause the domain wall to sit in a local energy minimum, requiring a larger activation energy (thermal) or external electric field to unpin the wall.[23] During this study, the temporal effects were probed. The BTO lamella was exposed for over 1 hour at 80°C in each environment. In every case, the domain structure did not change.[24]

Refences

[1] Climate In situ TEM Gas & Heating, https://denssolutions.com/products/climate/, Nov 11, 2022

[2] L. J. McGilly, Queen's University Belfast, 2012.

[3] R. G. McQuaid, Queen's University Belfast, 2012.

[4] J. Chrosch, E. K. H. Salje, *Journal of Applied Physics* **1999**, 85, 722.

[5] S. Bueble, K. Knorr, E. Brecht, W. W. Schmahl, *Surface Science* **1998**, 400, 345.

[6] K. A. Müller, W. Berlinger, F. Waldner, *Physical Review Letters* **1968**, 21, 814.

[7] J. J. R. Scott, B. Casals, K.-F. Luo, A. Haq, D. Mariotti, E. K. H. Salje, M. Arredondo, *Scientific Reports* **2022**, 12, 14818.

[8] Z. Wang, M. Reticcioli, Z. Jakub, I. Sokolović, M. Meier, L. A. Boatner, M. Schmid, G. S. Parkinson, U. Diebold, C. Franchini, M. Setvin, *Science Advances* **2022**, 8, eabq1433.

[9] C. Yue, X. Lu, J. Zhang, F. Huang, J. Zhu, *Physical Review B* **2019**, 100, 245432.

[10] T. L. Wan, J. Shang, Y. Gu, L. Kou, *Advanced Materials Technologies* **2022**, 7, 2100463.

[11] Y. Yun, E. I. Altman, *Journal of the American Chemical Society* **2007**, 129, 15684.

[12] S. Habicht, R. J. Nemanich, A. Gruverman, *Nanotechnology* **2008**, 19, 495303.

[13] J. Shin, V. B. Nascimento, G. Geneste, J. Rundgren, E. W. Plummer, B. Dkhil, S. V. Kalinin, A. P. Baddorf, *Nano Letters* **2009**, 9, 3720.

[14] A. N. Morozovska, E. A. Eliseev, I. S. Vorotiahin, M. V. Silibin, S. V. Kalinin, N. V. Morozovsky, *Acta Materialia* **2018**, 160, 57.

[15] K. Yasui, K. Kato, *The Journal of Physical Chemistry C* **2013**, 117, 19632.

[16] Z. Dohnálek, J. Kim, O. Bondarchuk, J. M. White, B. D. Kay, *The Journal of Physical Chemistry B* **2006**, 110, 6229.

[17] R. Otero, *Dyanmics of Organic Molecules at Solid Surfaces*, Elsevier, **2018**.

[18] J. J. Segura, N. Domingo, J. Fraxedas, A. Verdaguer, *Journal of Applied Physics* **2013**, 113, 187213.

[19] Z. Liu, Y. Song, A. Rajappan, E. N. Wang, D. J. Preston, *Langmuir* **2022**, 38, 1252.

[20] I. Spasojevic, G. Sauthier, J. M. Caicedo, A. Verdaguer, N. Domingo, *Applied Surface Science* **2021**, 565, 150288.

[21] X. Wang, T. Xu, F. Xuan, C. Chen, T. Shimada, T. Kitamura, *Journal of Applied Physics* **2019**, 126, 174107.
[22] Y. Noguchi, H. Matsuo, Y. Kitanaka, M. Miyayama, *Scientific Reports* **2019**, 9, 4225.
[23] T. Rojac, M. Kosec, B. Budic, N. Setter, D. Damjanovic, *Journal of Applied Physics* **2010**, 108, 074107.
[24] T. O'Reilly, Queen's University Belfast, 2022.
